# Association between the combined effects of *GSTM1* present/null and *CYP1A1* MspI polymorphisms with lung cancer risk: an updated meta-analysis

**DOI:** 10.1042/BSR20202275

**Published:** 2020-09-29

**Authors:** Wen-Ping Zhang, Xiao-Feng He, Xiang-Hua Ye

**Affiliations:** 1 Department of Cardiothoracic Surgery, Heping Hospital Affiliated to Changzhi Medical College, Changzhi 046000, China; 2 Department of Science and Education, Heping Hospital Affiliated to Changzhi Medical College, Shanxi, Changzhi 046000, China; 3 Department of Radiotherapy, First Affiliated Hospital, Zhejiang University School of Medicine, Hangzhou, China

**Keywords:** CYP1A1, GSTM1, Lung cancer, Meta-analysis, polymorphism

## Abstract

**Background:** Many studies have been performed to explore the combined effects of glutathione-S-transferase M1 (*GSTM1*) present/null and cytochrome P4501A1 (*CYP1A1*) MspI polymorphisms with lung cancer (LC) risk, but the results are contradictory. Two previous meta-analyses have been reported on the issue in 2011 and 2014. However, several new articles since then have been published. In addition, their meta-analyses did not valuate the credibility of significantly positive results.

**Objectives:** We performed an updated meta-analysis to solve the controversy following the Preferred Reporting Items for Systematic Reviews and Meta-Analyses guidelines.

**Methods:** False-positive report probability (FPRP), Bayesian false discovery probability (BFDP), and the Venice criteria were used to verify the credibility of meta-analyses.

**Results:** Twenty-three publications including 5734 LC cases and 7066 controls met the inclusion criteria in the present study. A significantly increased risk of LC was found in overall analysis, Asians and Indians. However, all positive results were considered as ‘less-credible’ when we used the Venice criteria, FPRP, and BFDP test to assess the credibility of the positive results.

**Conclusion:** These positive findings should be interpreted with caution and results indicate that significant associations may be less-credible, there are no significantly increased LC risk between the combined effects of *GSTM1* present/null and *CYP1A1* MspI polymorphisms.

## Background

Lung cancer (LC) is one of the most common malignancies and it is the leading cause of cancer deaths in both men and women [1–3]. It is an extremely complex disease because it is the result of the combined effects of genes, environment, and lifestyle [4–6]. As an example, smoking has been confirmed to be associated with increased LC risk [7]. However, not all smokers will get LC, therefore, other factors, such as genetic susceptibility, may play an important role in LC susceptibility [8,9].

Glutathione-S-transferase M1 (*GSTM1*) and cytochrome P4501A1 (*CYP1A1*) have been reported to be involved in the detoxification and bioactivation of chemical carcinogen in habitual smokers, which might lead to LC susceptibility [10,11]. The above two genes play an important role in the metabolism of polycyclic aromatic hydrocarbons (PAHs) [12]. *CYP1A1* including two genetic polymorphisms has been reported: one is IIe462Val (CYP1A1*2C) polymorphism and the other is MspI (CYP1A1*2A) polymorphism [13,14], which may result in an increased activity. *GSTM1* gene shows deletion polymorphisms (null genotype) [15], which cause the absence of expression and enzyme activity loss [16] and is located on chromosome 1 (1p13.3) [17]. As the preservation of genomic integrity is essential in the prevention of tumor initiation and progression, mutations and variations, especially in genes of enzymes in carcinogen metabolism, may play a role in the genetic predisposition to cancer. Therefore, genetic polymorphisms leading to altered activity in phase I enzymes may cause variations in the levels of DNA damage and cancer susceptibility [12].

Two large-scale meta-analysces [18,19] have been published in 2011 and 2014 that confirmed the combined effects of CYP1A1 MspI and *GSTM1* present/null genotypes to be significant risk factors for LC. However, several new articles have been published. Moreover, results of previous original studies [20–47] on the combined effects of the two genes were inconsistent or even contradictory, and individual studies may be underpowered to detect the effect of polymorphism on the susceptibility of LC. Furthermore, previous two meta-analyses did not evaluate the credibility of significantly positive results on the issue. Hence, the association of this issue remains unknown. It is very important to identify the genotype distribution for predicting the risk of LC and understanding the pathogenesis of LC. Hence, an updated meta-analysis was performed to provide a more precise evaluation on such association. In addition, to minimize random errors and strengthen the robustness of the results, we performed a trial sequential analysis (TSA). Moreover, we used false-positive report probability (FPRP) [48], Bayesian false discovery probability (BFDP) [49], and the Venice criteria [50,51] to evaluate the credibility of significantly positive results in the present study.

## Materials and methods

The present meta-analysis was performed by the Preferred Reporting Items for Systematic Reviews and Meta-Analyses guidelines [52].

### Search strategy

PubMed, China National Knowledge Infrastructure (CNKI), and Wan Fang were used to search eligible studies. The latest date was 8 May 2020. We used the following keywords: (*GSTM1* OR Glutathione S-transferase M1 OR Glutathione S-transferase Mu 1) AND (Cytochrome P450 1A1 OR *CYP1A1*) AND lung. The corresponding authors were contacted when some studies were not available in full-text. If necessary, some reference lists of selected articles were carefully examined by hand searching.

### Inclusion and exclusion criteria

Publications will be selected if they met the following inclusion criteria: (1) publications regarding the combined effects of *GSTM1* present/null and *CYP1A1* MspI polymorphisms with LC risk; (2) case–control or cohort studies; (3) selecting the maximum sample size when data of one study was duplicated with another study; (4) providing the combined genotype data or ORs and their 95% CIs. Articles will be excluded if they met the following criteria: (1) original data not shown, (2) only cases, and (3) reviews, conference abstracts, letters and editorials.

### Data extraction

Data were independently extracted by two authors. Each eligible study includes the following data: (1) first author’s name, (2) publication year, (3) country, (4) ethnicity, (5) source of controls, (6) matching, (7) sample size, and (8) genotype distribution of cases and controls. The individuals from China and Japan were regarded as ‘Asians’, from Spain, Russia, Greece and other Western countries were regarded as ‘Caucasian’, and from India were as ‘Indians’. If one study did not state race or sample included several races, ‘Mixed populations’ was used.

### Quality score evaluation

The quality of the selected studies was evaluated independently by two authors. Table 1 lists the literature quality assessment criteria. The criteria were designed by previous meta-analyses about molecular epidemiology studies [53,54]. The highest value was 21 score in the quality assessment; studies scoring ≥12 were considered as high quality. Inconsistent scores were adjudicated by a third author.

**Table 1:** Scale for quality assessment of molecular association studies of LC

Criterion	Score
Source of case	
Selected from population or cancer registry	3
Selected from hospital	2
Selected from pathology archives, but without description	1
Not described	0
Source of control	
Population-based	3
Blood donors or volunteers	2
Hospital-based	1
Not described	0
Ascertainment of cancer	
Histological or pathological confirmation	2
Diagnosis of LC by patient medical record	1
Not described	0
Ascertainment of control	
Controls were tested to screen out LC	2
Controls were subjects who did not report LC, no objective testing	1
Not described	0
Matching	
Controls matched with cases by age	1
Not matched or not described	0
Genotyping examination	
Genotyping done blindly and quality control	2
Only genotyping done blindly or quality control	1
Unblinded and without quality control	0
Specimens used for determining genotypes	
Blood cells or normal tissues	1
Tumor tissues or exfoliated cells of tissue	0
HWE	
HWE in the control group	1
Hardy–Weinberg disequilibrium in the control group	0
Association assessment	
Assess association between genotypes and breast cancer with appropriate statistics and adjustment for confounders	2
Assess association between genotypes and breast cancer with appropriate statistics without adjustment for confounders	1
Inappropriate statistics used	0
Total sample size	
>1000	3
500–1000	2
200–500	1
<200	0

Abbreviation: HWE, Hardy–Weinberg equilibrium.

### TSA

TSA was conducted as described by a previous meta-analysis [55]. Briefly, α (type I error) and β (type II error) adopted a level of significance of 0.05 and 0.2, respectively. Information size was calculated using accrued information size (AIS), and TSA monitoring boundaries were also built.

### Credibility analysis

To evaluate the credibility of statistically significant results, FPRP, BFDP, and the Venice criteria were applied. Significant association was considered as ‘noteworthy’ when the results of FPRP and BRDP were less than 0.2 and 0.8, respectively. Concerning the Venice criteria, we assessed the criteria of amount of evidence by statistical power: A: ≥80%, B: 50–79%, and C: <50%. For replication, we applied the *I*^2^ recommended by Ioannidis et al. [50]: A: <25%, B: 25–50%, and C: >50%. For avoiding biases, we considered using the criteria proposed by Ioannidis et al. [50].

### Statistical analysis

The association between the combined effects of the *GSTM1* present/null and *CYP1A1* MspI polymorphisms and LC risk was assessed using pooled crude ORs and 95% CIs. The following eight genetic models were used: *GSTM1* null/*CYP1A1* m1/m1 vs. *GSTM1* present/*CYP1A1* m1/m1, *GSTM1* present/*CYP1A1* m1/m2 vs. *GSTM1* present/*CYP1A1* m1/m1, *GSTM1* null/*CYP1A1* m1/m2 vs. *GSTM1* present/*CYP1A1* m1/m1, *GSTM1* present/*CYP1A1* m2/m2 vs. *GSTM1* present/*CYP1A1* m1/m1, *GSTM1* null/*CYP1A1* m1/m1 vs. *GSTM1* present/*CYP1A1* m1/m1, *GSTM1* present/*CYP1A1* m* vs. *GSTM1* present/*CYP1A1* m1/m1, *GSTM1* null/*CYP1A1* m* vs. *GSTM1* present/*CYP1A1* m1/m1, and all risk genotypes vs. *GSTM1* present/*CYP1A1* m1/m1. Heterogeneity was estimated by the Cochran’s *Q* [56] and *I*^2^ value [57]. Significant heterogeneity was considered if *P*<0.10 and/or *I*^2^ > 50%. A fixed-effects model (Mantel–Haenszel method) was applied if no heterogeneity [58]; otherwise, a random-effects model (DerSimonian and Laird method) was used [59]. Hardy–Weinberg equilibrium (HWE) was detected according to chi-square goodness-of-fit test, and significant deviation was considered in controls when *P*<0.05. Sensitivity analysis was performed by the following methods: (1) each time that a single study was removed, and (2) a dataset was used that comprised only high-quality and controls in HWE studies. Begg’s funnel [60] and Egger’s test [61] were used to assess the publication bias. In addition, we estimated the heterogeneity source by meta-regression analysis. All statistical analyses were calculated using STATA version 12.0 (STATA Corporation, College Station, TX).

## Results

### Study characteristics

**Figure 1:**
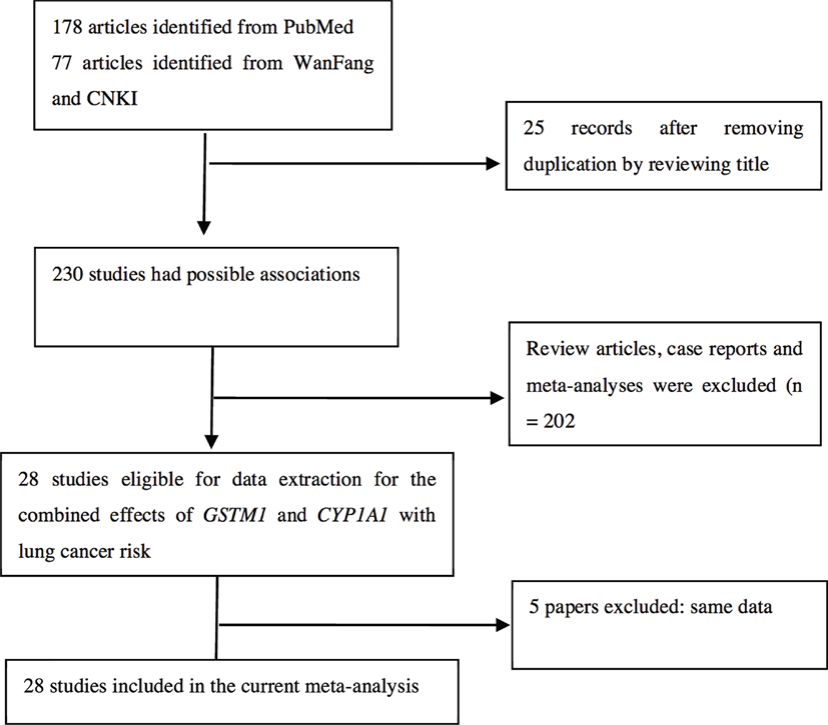
Flow diagram for identifying and including studies in the current meta-analysis

A total of 178, 35, and 42 studies were identified from PubMed, CNKI, and Wanfang databases (Figure 1), respectively. In total, 227 studies were excluded when titles and abstracts were appraised by review articles, case reports, and meta-analyses. In addition, the data of the five publications [21,31,37,38,42] were included in another four articles [27,35,45,58]. Therefore, 23 publications were included in the current study, as shown in Table 2. Of these publications, four studies were from Caucasians, four were from Indian populations, thirteen were from Asians, and two were from mixed populations. Furthermore, there were ten high-quality studies and thirteen low-quality studies (as also shown in Table 2). Table 3 lists the genotype frequencies of the combined effects of *GSTM1* present/null and *CYP1A1* MspI polymorphisms with LC risk.

**Table 2:** General characteristics of studies included in pooling gene effects

First author/year	Country	Ethnicity	Sample size	SC	Matching	Quality score
Peddireddy et al. (2016) [20]	India	Indian	246/250	PB	Age and sex	15
Girdhar et al. (2016) [44]	India	Indian	149/185	HB	Age and sex	10
López-Cima et al. (2012) [22]	Spain	Caucasian	789/789	HB	Age and sex	17
Li et al. (2011) [45]	China	Asian	103/138	HB	ND	11
Jin et al. (2010) [25]	China	Asian	150/150	HB	Age and sex	13
Zhu et al. (2010) [24]	China	Asian	160/160	HB	ND	11
Chang et al. (2009) [23]	China	Asian	263/263	HB	Age and sex	14
Shah et al. (2008) [28]	India	Indian	200/200	HB	Age and sex	13
Xia et al. (2008) [46]	China	Asian	58/116	HB	Age	11
Hou et al. (2008) [47]	China	Asian	77/77	HB	Sex	9
Gu et al. (2007) [27]	China	Asian	279/684	HB	ND	9
Belogubova et al. (2006) [32]	Russia	Caucasian	141/450	HB	ND	12
Wang et al. (2006) [36]	China	Asian	91/91	HB	Age	6
Sreeja et al. (2005) [34]	India	Indian	146/146	HB	Age and sex	14
Wang et al. (2004) [39]	China	Asian	91/91	HB	Age	8
Vineis et al. (2004) [35]	Multiple	Caucasian	1466/1488	HB/PB	ND	13
Dialyna et al. (2003) [40]	Greece	Caucasian	122/178	HB	ND	10
Cheng et al. (2000) [41]	China	Asian	73/33	HB	ND	7
Song et al. (2000) [33]	China	Asian	167/391	PB	Age and sex	15
Le Marchand et al. (1998) [29]	U.S.A.	Mixed	341/456	PB	Age and sex	18
Hong et al. (1998) [30]	Korea	Asian	85/63	HB	ND	8
Garcia-Closas et al. (1997) [26]	U.S.A.	Mixed	442/412	HB	ND	12
Kihara and Noda (1995) [43]	Japan	Asian	95/255	HB	ND	11

Abbreviations: CR, cancer registry; HB, hospital-based study; HP, healthy population; ND, not described; PB, population-based study.

**Table 3:** Genotype frequencies of the combined effects of *GSTM1* present/null and *CYP1A1* MspI polymorphisms and LC risk

First author/year	Present/m1/m1		Null/m1/m1		Present/m1/m2		Null/m1/m2		Present/m2/m2		Null/ m2/m2		Present/m*		Null/m*		HWE for CYP1A1
	Case	Control	Case	Control	Case	Control	Case	Control	Case	Control	Case	Control	Case	Control	Case	Control	
Peddireddy et al. (2016) [20]	74	133	31	40	84	44	32	19	21	12	4	2	105	56	36	21	Y
Girdhar et al. (2016) [44]	71	101	NA	NA	NA	NA	71	56	NA	NA	15	9	NA	NA	86	65	N
López-Cima et al. (2012) [22]	283	286	90	70	NA	NA	NA	NA	NA	NA	NA	NA	308	332	89	84	Y
Li et al. (2011) [45]	12	40	15	33	24	33	39	21	3	4	9	7	27	37	48	28	Y
Jin et al. (2010) [25]	27	40	52	40	NA	NA	NA	NA	NA	NA	NA	NA	28	31	43	39	Y
Zhu et al. (2010) [24]	29	38	26	30	23	36	45	30	15	14	22	12	38	50	67	42	Y
Chang et al. (2009) [23]	82	100	80	88	NA	NA	NA	NA	NA	NA	NA	NA	29	37	72	38	Y
Shah et al. (2008) [28]	60	103	46	34	41	44	32	12	10	5	11	2	51	49	43	14	Y
Xia et al. (2008) [46]	7	21	10	19	20	30	16	28	1	6	4	12	21	36	20	40	Y
Hou et al. (2008) [47]	16	19	19	21	15	4	19	20	1	6	7	7	16	10	26	27	N
Gu et al. (2007) [27]	34	154	57	149	NA	NA	NA	NA	NA	NA	NA	NA	81	205	107	176	Y
Belogubova et al. (2006) [32]	49	183	55	174	17	48	18	42	1	0	1	3	18	48	19	45	Y
Wang et al. (2006) [36]	16	19	16	16	10	9	13	21	9	12	27	14	19	21	40	35	N
Sreeja et al. (2005) [34]	48	68	23	24	NA	NA	NA	NA	NA	NA	NA	NA	52	40	23	14	Y
Wang et al. (2004) [39]	19	28	13	25	7	19	16	27	9	18	27	21	16	37	43	48	N
Vineis et al. (2004) [35]	560	573	653	640	102	112	127	145	8	6	16	12	110	118	143	157	Y
Dialyna et al. (2003) [40]	40	57	49	73	17	23	11	22	2	2	3	1	19	25	14	23	Y
Cheng et al. (2000) [41]	35	12	24	11	NA	NA	NA	NA	NA	NA	NA	NA	4	4	10	6	Y
Song et al. (2000) [33]	23	95	10	66	NA	NA	NA	NA	NA	NA	NA	NA	74	146	44	79	Y
Le Marchand et al. (1998) [29]	50	101	76	147	NA	NA	NA	NA	NA	NA	NA	NA	50	81	59	121	N
Hong et al. (1998) [30]	14	11	21	15	21	18	22	16	3	1	4	2	24	19	26	18	Y
Garcia-Closas et al. (1997) [26]	174	155	195	182	38	32	35	43	NA	NA	NA	NA	NA	NA	NA	NA	Y
Kihara and Noda (1995) [43]	18	48	18	64	21	54	24	51	3	25	13	16	24	79	37	67	Y

Abbreviation: NA, not available.

### Meta-analysis results

**Figure 2:**
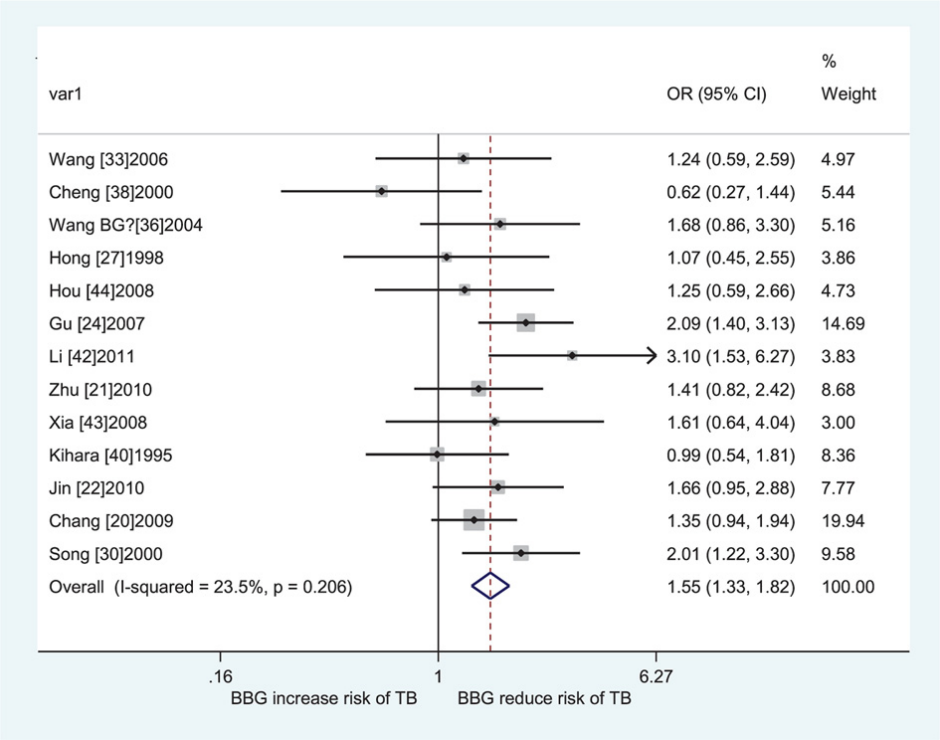
Forest plot of the association between the combined effects of *GSTM1* present/null and *CYP1A1* MspI polymorphisms with LC risk in Asians (all risk genotypes vs. *GSTM1* present/*CYP1A1* m1/m1)

**Figure 3:**
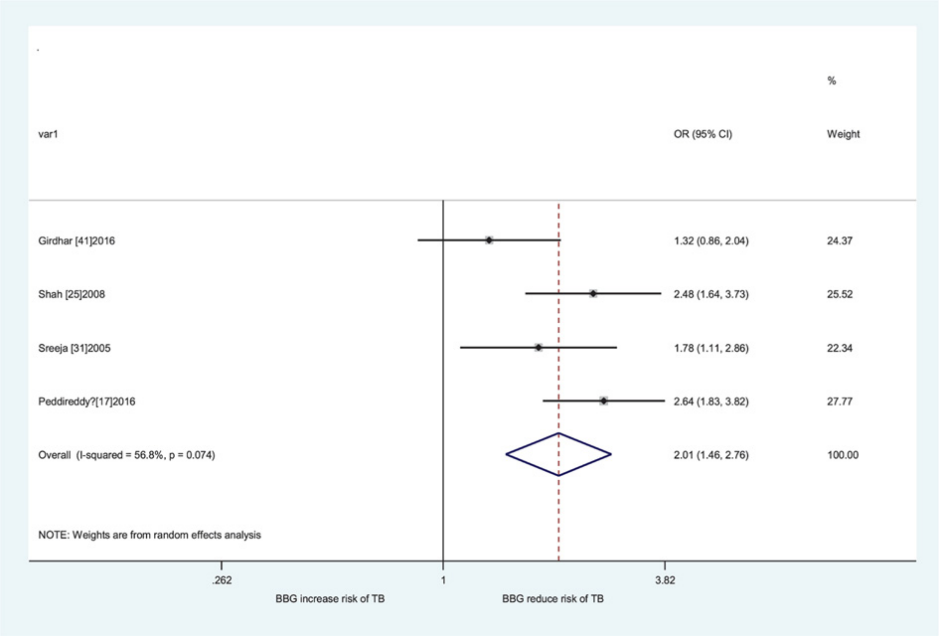
Forest plot of the association between the combined effects of *GSTM1* present/null and *CYP1A1* MspI polymorphisms with LC risk in Indians (all risk genotypes vs. *GSTM1* present/*CYP1A1* m1/m1)

The results of pooled analyses were shown in Table 4. The individuals carrying the *GSTM1* null/*CYP1A1* m1/m1, *GSTM1* present/*CYP1A1* m1/m2, *GSTM1* null/*CYP1A1* m1/m2, *GSTM1* null/*CYP1A1* m2/m2, *GSTM1* present/*CYP1A1* m*, *GSTM1* null/*CYP1A1* m*, and all risk genotypes, the pooled ORs with their 95% CIs for all populations were 1.13 (1.03–1.24), 1.36 (1.01–1.83), 1.48 (1.07–2.06), 2.16 (1.62–2.89), 1.33 (1.08–1.63), 1.69 (1.32–2.16), and 1.43 (1.22–1.67) when compared with *GSTM1* present/*CYP1A1* m1/m1, respectively. Then, we performed a subgroup analysis by ethnicity, significantly increased LC risk was observed in Asians (*GSTM1* null/*CYP1A1* m2/m2 vs. *GSTM1* present/*CYP1A1* m1/m1: OR = 2.05, 95% CI = 1.42–2.95; *GSTM1* present/*CYP1A1* m* vs. *GSTM1* present/*CYP1A1* m1/m1: OR = 1.32, 95% CI = 1.09–1.61; *GSTM1* null/*CYP1A1* m* vs. *GSTM1* present/*CYP1A1* m1/m1: OR = 1.85, 95% CI = 1.44–2.38; all risk genotypes vs. *GSTM1* present/*CYP1A1* m1/m1: OR = 1.55, 95% CI = 1.33–1.82, Figure 2) and Indians (*GSTM1* null/*CYP1A1* m1/m1 vs. *GSTM1* present/*CYP1A1* m1/m1: OR = 1.68, 95% CI = 1.20–2.35; *GSTM1* present/*CYP1A1* m1/m2 vs. *GSTM1* present/*CYP1A1* m1/m1: OR = 2.37, 95% CI = 1.12–5.01; *GSTM1* null/*CYP1A1* m1/m2 vs. *GSTM1* present/*CYP1A1* m1/m1: OR = 2.76, 95% CI = 1.60–4.75; *GSTM1* present/*CYP1A1* m2/m2 vs. *GSTM1* present/*CYP1A1* m1/m1: OR = 3.24, 95% CI = 1.72–6.08; *GSTM1* null/*CYP1A1* m2/m2 vs. *GSTM1* present/*CYP1A1* m1/m1: OR = 3.59, 95% CI = 1.82–7.09; *GSTM1* present/*CYP1A1* m* vs. *GSTM1* present/*CYP1A1* m1/m1: OR = 2.28, 95% CI = 1.48–3.51; *GSTM1* null/*CYP1A1* m* vs. *GSTM1* present/*CYP1A1* m1/m1: OR = 3.44, 95% CI = 2.34–5.06; all risk genotypes vs. *GSTM1* present/*CYP1A1* m1/m1: OR = 2.01, 95% CI = 1.46–2.77, Figure 3).

**Table 4:** The results of the pooled analysis between the combined effects of *GSTM1* present/null and *CYP1A1* MspI and LC risk

Variable	*n*	Cases/controls	Test of association		Test of heterogeneity		Prior probability of 0.001			Venice criteria
			OR (95% CI)	*P*	*P*_h_	*I*^2^ (%)	Power	FPRP	BFDP	
*GSTM1* null/*CYP1A1* m1/m1 vs. *GSTM1* present/*CYP1A1* m1/m1										
Overall	22	3249/4245	**1.13 (1.03, 1.24)**	0.013	0.401	4.5	1.000	0.908	0.998	AAB
Ethnicity										
Asian	13	693/122	1.18 (0.97, 1.44)	0.101	0.534	0.0	-	-	-	-
Indian	3	282/402	**1.68 (1.20, 2.35)**	0.002	0.337	8.2	0.254	0.906	0.981	CAB
Caucasian	4	1779/2056	1.08 (0.95, 1.24)	0.236	0.665	0.0	-	-	-	-
Mixed	2	495/585	0.98 (0.77, 1.26)	0.883	0.738	0.0	-	-	-	-
*GSTM1* present/*CYP1A1* m1/m2 vs. *GSTM1* present/*CYP1A1* m1/m1										
Overall	14	1,528/1,934	1.36 (1.01, 1.83)*	0.045	0.001	62.6	0.741	0.983	0.998	BCB
Ethnicity										
Asian	8	272/427	1.27 (0.92, 1.74)	0.143	0.127	37.9	-	-	-	-
Indian	2	259/324	2.37 (1.12, 5.01)*	0.024	0.034	77.8	0.116	0.995	0.997	CCB
Caucasian	3	785/996	1.00 (0.77, 1.28)	0.973	0.612	0.0	-	-	-	-
Mixed	1	212/187	1.06 (0.63, 1.78)	0.83	-	-	-	-	-	-
*GSTM1* null/*CYP1A1* m1/m2 vs. *GSTM1* present/*CYP1A1* m1/m1										
Overall	15	1679/2082	1.48 (1.07, 2.06)*	0.019	<0.001	73.5	0.532	0.974	0.997	BCB
Ethnicity										
Asian	8	325/438	1.49 (0.93, 2.38)*	0.095	0.021	57.4	-	-	-	-
Indian	3	340/424	2.76 (1.60, 4.75)*	<0.001	0.088	58.8	0.014	0.947	0.911	CCB
Caucasian	3	805/1022	0.95 (0.75, 1.20)	0.657	0.198	38.2	-	-	-	-
Mixed	1	209/198	0.73 (0.44, 1.19)	0.204	-	-	-	-	-	-
*GSTM1* present/*CYP1A1* m2/m2 vs. *GSTM1* present/*CYP1A1* m1/m1										
Overall	13	1000/1384	1.32 (0.82, 2.14)*	0.255	0.067	40.0	-	-	-	-
Ethnicity										
Asian	8	175/310	0.83 (0.53, 1.29)	0.411	0.354	9.8	-	-	-	-
Indian	2	165/253	**3.24 (1.72, 6.08)**	<0.001	0.899	0.0	0.008	0.968	0.928	CAB
Caucasian	3	660/821	1.65 (0.68, 3.99)	0.271	0.474	0.0	-	-	-	-
*GSTM1* null/*CYP1A1* m2/m2 vs. *GSTM1* present/*CYP1A1* m1/m1										
Overall	14	1148/1494	**2.16 (1.62, 2.89)**	<0.001	0.735	0.0	0.007	0.030	0.014	CAB
Ethnicity										
Asian	8	244/315	**2.05 (1.42, 2.95)**	<0.001	0.831	0.0	0.046	0.705	0.791	CAB
Indian	3	235/350	**3.59 (1.82, 7.09)**	<0.001	0.061	15.6	0.006	0.975	0.934	CAB
Caucasian	3	669/829	1.52 (0.77, 3.00)	0.224	0.642	0.0	-	-	-	-
*GSTM1* present/*CYP1A1* m* vs. *GSTM1* present/*CYP1A1* m1/m1										
Overall	21	2610/3590	1.33 (1.08, 1.63)*	0.006	<0.001	61.6	0.877	0.872	0.993	ACB
Ethnicity										
Asian	13	733/1,337	**1.32 (1.09, 1.61)**	0.005	0.121	32.7	0.896	0.873	0.993	AAB
Indian	3	390/449	2.28 (1.48, 3.51)*	<0.001	0.102	56.1	0.029	0.864	0.863	CCB
Caucasian	4	1387/1622	0.98 (0.83, 1.15)	0.777	0.682	0.0	-	-	-	-
Mixed	1	100/182	1.25 (0.77,2.03)	0.376	-	-	-	-	-	-
*GSTM1* null/*CYP1A1* m* vs. *GSTM1* present/*CYP1A1* m1/m1										
Overall	21	2505/3251	1.69 (1.32, 2.16)*	<0.001	<0.001	71.3	0.170	0.140	0.516	CCB
Ethnicity										
Asian	13	915/1,268	1.85 (1.44, 2.38)*	<0.001	0.069	39.8	0.051	0.032	0.077	CAB
Indian	3	284/353	**3.44 (2.34, 5.06)**	<0.001	0.266	24.4	<0.001	0.027	<0.001	CAB
Caucasian	4	1197/1408	1.01 (0.84, 1.22)	0.887	0.456	0.0	-	-	-	-
Mixed	1	109/222	0.99 (0.62, 1.56)	0.949	-	-	-	-	-	-
All risk genotypes vs. *GSTM1* present/*CYP1A1* m1/m1										
Overall	23	5734/7066	1.43 (1.22, 1.67)*	<0.001	<0.001	68.5	0.727	0.008	0.243	BCC
Ethnicity										
Asian	13	1692/2512	**1.55 (1.33, 1.82)**	<0.001	0.206	23.5	0.344	<0.001	0.006	CAB
Indian	4	741/781	**2.01 (1.46, 2.77)**	<0.001	0.074	56.8	0.037	0.350	0.448	CCB
Caucasian	4	2518/2905	1.03 (0.92, 1.15)	0.584	0.717	0.0	-	-	-	-
Mixed	2	783/868	-	-	0.015	83.3	-	-	-	-

*Random-effects model was used in the pooled data. Note: The bold values indicate significant results.

### Heterogeneity and sensitivity analyses

A meta-regression analysis was performed to explore the heterogeneity source. Current study indicated that ethnicity, source of controls, type of controls, matching, HWE, quality score, and sample size were not heterogeneity source. The results did not change if a single study was deleted each time (results not shown). In addition, the results also did not change when studies only including controls in HWE and high quality were pooled, as shown in Table 5.

**Table 5:** The results of sensitivity analysis between the combined effects of *GSTM1* present/null and *CYP1A1* MspI and LC risk

Variable	*n*	Cases/controls	Test of association		Test of heterogeneity		Prior probability of 0.001			Venice criteria
			OR (95% CI)	*P*	*P*_h_	*I*^2^ (%)	Power	FPRP	BFDP	
*GSTM1* null/*CYP1A1* m1/m1 vs. *GSTM1* present/*CYP1A1* m1/m1										
Overall	10	2615/3094	**1.20 (1.01, 1.42)**	0.035	0.088	40.5	0.995	0.971	0.999	AAB
Ethnicity										
Asian	3	274/429	1.15 (0.67, 1.96)	0.622	0.095	57.6	-	-	-	-
Indian	3	282/402	**1.68 (1.20, 2.35)**	0.002	0.337	8.2	0.254	0.906	0.981	CAB
Caucasian	3	1690/1926	1.09 (0.95, 1.25)	0.002	0.337	8.2	-	-	-	-
Mixed	1	369/337	0.95 (0.71, 1.28)	0.758	-	-	-	-	-	-
*GSTM1* present/*CYP1A1* m1/m2 vs. *GSTM1* present/*CYP1A1* m1/m1										
Overall	5	1199/1427	-	-	<0.001	82.6	-	-	-	-
Ethnicity										
Indian	2	259/324	-	-	0.034	77.8	-	-	-	-
Caucasian	2	728/916	0.99 (0.76, 1.29)	0.933	0.327	0.0	-	-	-	-
Mixed	1	212/187	1.05 (0.63, 1.78)	0.831	-	-	-	-	-	-
*GSTM1* null/*CYP1A1* m1/m2 vs. *GSTM1* present/*CYP1A1* m1/m1										
Overall	5	1161/1408	-	-	<0.001	86.8	-	-	-	-
Ethnicity										
Indian	2	198/267	**3.63 (2.25, 5.86)**	<0.001	0.404	0.0	<0.001	0.469	0.020	CAB
Caucasian	2	754/943	1.11 (0.64, 1.92)	0.707	0.099	63.3	-	-	-	-
Mixed	1	209/198	0.73 (0.44, 1.19)	0.204	-	-	-	-	-	-
*GSTM1* present/*CYP1A1* m2/m2 vs. *GSTM1* present/*CYP1A1* m1/m1										
Overall	4	783/1015	**2.68 (1.58, 4.56)**	<0.001	0.448	0.0	0.016	0.945	0.916	CAB
Ethnicity										
Indian	2	165/253	**3.24 (1.72, 6.08)**	<0.001	0.899	0.0	0.008	0.968	0.928	CAB
Caucasian	2	618/762	1.70 (0.63, 4.59)	0.292	0.225	32.2	-	-	-	-
*GSTM1* null/*CYP1A1* m2/m2 vs. *GSTM1* present/*CYP1A1* m1/m1										
Overall	4	775/1011	**2.26 (1.27, 4.04)**	0.006	0.135	46.0	0.083	0.986	0.991	CBB
Ethnicity										
Indian	2	149/240	**6.49 (2.11, 19.99)**	0.001	0.409	0.0	0.005	0.995	0.991	CAB
Caucasian	2	626/771	1.35 (0.66, 2.77)	0.410	0.941	0.0	-	-	-	-
*GSTM1* present/*CYP1A1* m* vs. *GSTM1* present/*CYP1A1* m1/m1										
Overall	10	2081/2620	-	-	<0.001	76.6	-	-	-	-
Ethnicity										
Asian	3	263/449	**1.44 (1.03, 2.01)**	0.034	0.140	49.1	0.595	0.982	0.998	BBB
Indian	3	390/449	**2.28 (1.49, 3.51)**	<0.001	0.102	56.1	0.029	0.864	0.863	CCB
Caucasian	3	1328/1540	0.97 (0.82, 1.15)	0.732	0.492	0.0	-	-	-	-
Mixed	1	100/182	1.25 (0.76, 2.03)	0.376	-	-	-	-	-	-
*GSTM1* null/*CYP1A1* m* vs. *GSTM1* present/*CYP1A1* m1/m1										
Overall	10	1827/2294	-	-	<0.001	79.7	-	-	-	-
Ethnicity										
Asian	3	291/391	**2.12 (1.53, 2.93)**	<0.001	0.668	0.0	0.018	0.228	0.204	CAB
Indian	3	284/353	**3.44 (2.34, 5.06)**	<0.001	0.266	24.4	<0.001	0.027	<0.001	CAB
Caucasian	3	1143/1328	1.02 (0.84, 1.24)	0.813	0.295	18.1	-	-	-	-
Mixed	1	109/222	0.99 (0.62, 1.56)	0.949	-	-	-	-	-	-
All risk genotypes vs. *GSTM1* present/*CYP1A1* m1/m1										
Overall	10	4010/4539	-	-	<0.001	81.6	-	-	-	-
Ethnicity										
Asian	3	580/804	**1.59 (1.23, 2.05)**	<0.001	0.442	0.0	0.327	0.515	0.911	CAB
Indian	3	592/596	**2.34 (1.85, 2.97)**	<0.001	0.413	0.0	<0.001	<0.001	<0.001	CAB
Caucasian	3	2396/2727	1.04 (0.92, 1.16)	0.553	0.527	0.0	-	-	-	-
Mixed	1	442/412	0.93 (0.71, 1.22)	0.601	-	-	-	-	-	-

*Random-effects model was used in the pooled data. Note: The bold values indicate significant results.

### Publication bias

**Figure 4:**
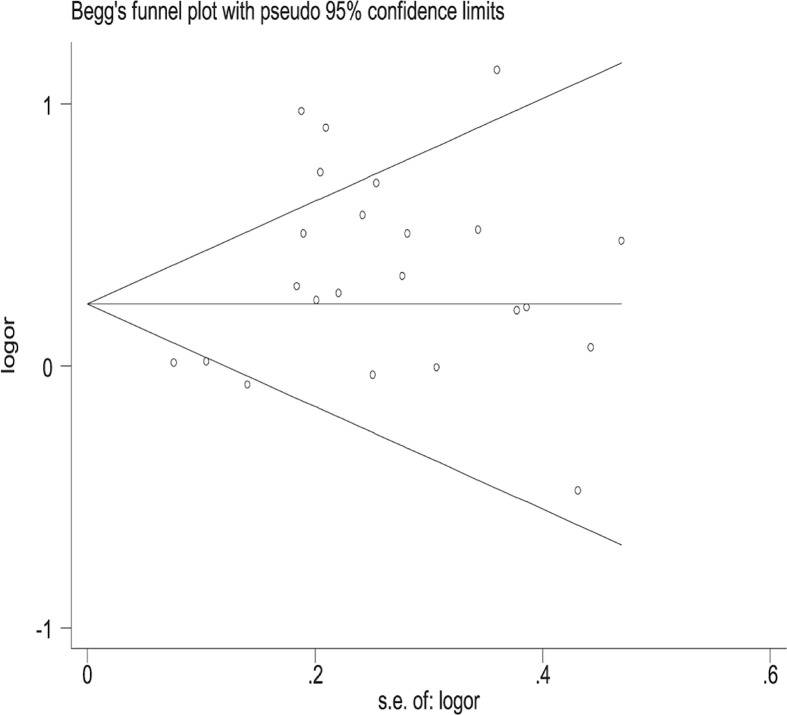
Begg’s funnel plot to assess publication bias on the combined effects of *GSTM1* and *CYP1A1* with LC risk in overall population (all risk genotypes vs. *GSTM1* present/*CYP1A1* m1/m1)

**Figure 5:**
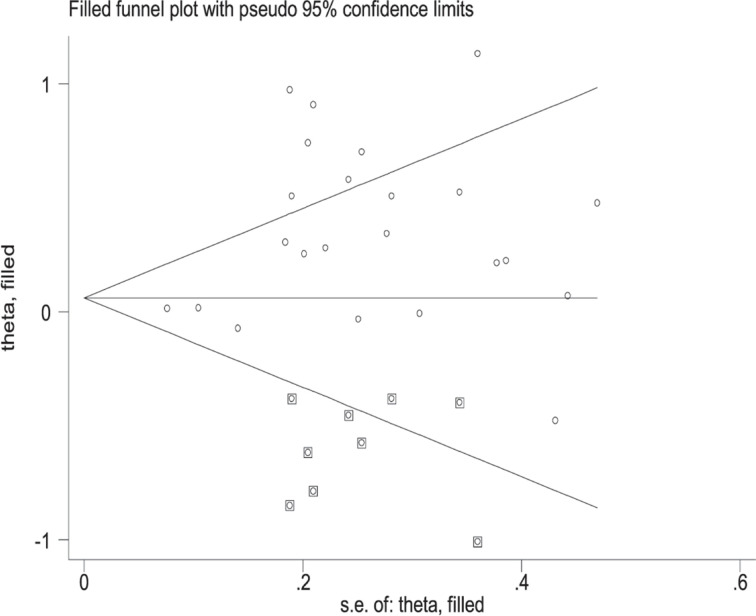
The Duval and Tweedie nonparametric ‘trim and fill’ method’s funnel plot of the combined effects of *GSTM1* and *CYP1A1* with LC risk (all risk genotypes vs. *GSTM1* present/*CYP1A1* m1/m1)

Obvious publication bias was found by Egger’s test in all risk genotypes vs. *GSTM1* present/*CYP1A1* m1/m1 (*P*=0.030) and Begg’s funnel plots (Figure 4). Results changed (all risk genotypes vs. *GSTM1* present/*CYP1A1* m1/m1: OR = 1.09, 95% CI: 0.91–1.30) in overall analysis after using the nonparametric ‘trim and fill’ method (Figure 5).

### TSA and credibility of the positive results

**Figure 6:**
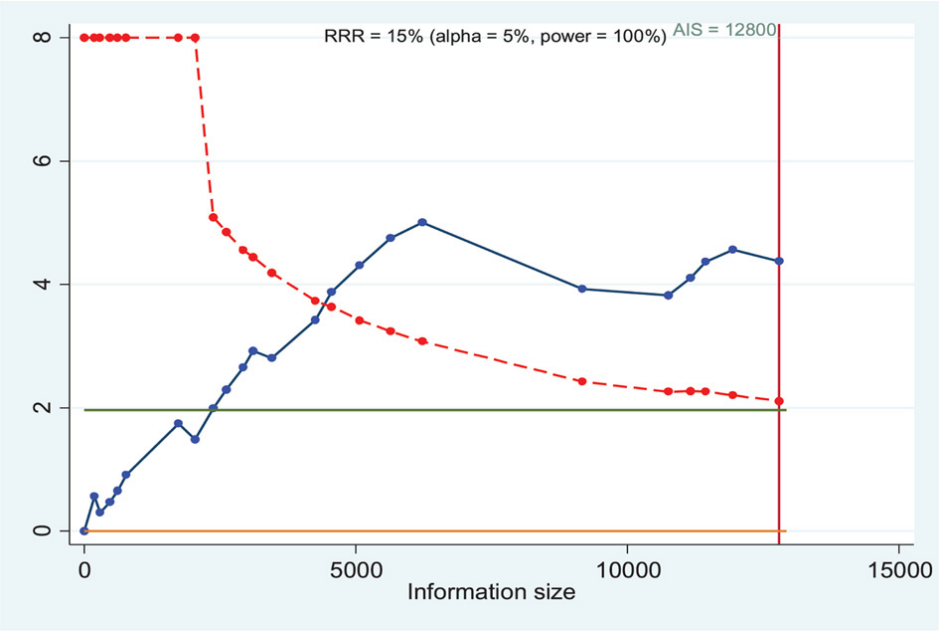
TSA for the combined effects of *GSTM1* present/null and *CYP1A1* MspI polymorphisms with LC risk in overall population (all risk genotypes vs. *GSTM1* present/*CYP1A1* m1/m1 model)

Figure 6 lists the TSA for the combined effects of *GSTM1* present/null and *CYP1A1* MspI polymorphisms with LC risk in overall population (all risk genotypes vs. *GSTM1* present/*CYP1A1* m1/m1 model). The result indicated the cumulative evidence is sufficient. Then, we applied FPRP, BFDP, and the Venice criteria to assess the credibility of statistically significant results. All positive results were considered as ‘less-credible’ (Tables 4 and 5).

## Discussion

In 1994, Alexandrie et al. [21] first investigated the combined effects between *GSTM1* present/null and *CYP1A1* MspI polymorphisms and LC risk. Since then, many studies have been published. However, the results of these studies were contradictory. In addition, two published meta-analyses did not assess the credibility of significantly positive results. Therefore, an updated meta-analysis was calculated to investigate the association between the combined effects of *GSTM1* present/null and *CYP1A1* MspI polymorphisms with LC risk.

In the present study, we observed that the individuals carrying *GSTM1* null/CYP1A1 m1/m1, *GSTM1* present/*CYP1A1* m1/m2, *GSTM1* null/*CYP1A1* m1/m2, *GSTM1* null/*CYP1A1* m2/m2, *GSTM1* present/*CYP1A1* m*, *GSTM1* null/*CYP1A1* m*, and all risk genotypes were associated with LC risk. In addition, statistically significant increased LC risk was also found in Asians and Indians. Moreover, when we restrained only high-quality and HWE studies, statistically significant increased LC risk still be observed in overall population, Asians, and Indians. Then, we performed a TSA in the present study and the results indicated that the cumulative evidence is sufficient. Actually, it may be common that the same polymorphism played different roles in cancer risk among different ethnic populations, because cancer is a complicated multigenetic disease, and different genetic backgrounds may contribute to the discrepancy [12]. Five [25,27,33,45,47] and three [20,28,34] studies indicated that the combined effects of *GSTM1* present/null and *CYP1A1* MspI polymorphisms with LC risk in Asians and Indians, respectively. However, eight different genetic models were used in this meta-analysis to explore the association. In this case, the *P*-value must be adjusted to make multiple comparisons clear [62]. In addition, a lot of evidence was required to ensure statistical power to reach more stringent levels of statistical significance or lower false-discovery rate for detecting associations, especially in molecular epidemiological studies [63]. Therefore, we used FPRP, BFDP, and the Venice criteria to assess the credibility of thees positive results, and found that all significant associations were considered as ‘less-credibility’.

Significant publication bias was found by Begg’s funnel plots and Egger’s test in all risk genotypes vs. *GSTM1* present/*CYP1A1* m1/m1 (*P*=0.030). Random error and bias were common in the studies with small sample sizes, and the results may be unreliable in molecular epidemiological studies. Furthermore, small sample studies were easier to accept if there was a positive report as they tend to yield false-positive results because they may be not rigorous and are often of low quality. Figure 4 indicates that the asymmetry of the funnel plot was caused by a study with low-quality small samples. In addition, at any case, the association between between the combined effects of *GSTM1* present/null and *CYP1A1* MspI polymorphisms with LC risk in Indians (*n*=4) and Caucasians (*n*=4) remain an open field, because the number of studies are considerably smaller than that needed for the achievement of robust conclusions [64]. Therefore, a huge population-based case–control study is required to confirm these associations in Indians and Caucasians.

Two meta-analyses have been published on the association between the combined effects of *GSTM1* present/null and *CYP1A1* MspI polymorphisms and LC risk. Li et al. [18] only examined seven studies (809 LC cases and 935 controls) and their meta-analysis indicated that the combined effects of *GSTM1* present/null and *CYP1A1* MspI polymorphisms were significantly associated with an increased LC risk. Li et al. [19] selected 21 studies including 3896 LC cases and 4829 controls for investigaton, and results were same as Li et al. [18]. However, their studies did not exclude the quality studies to further perform a meta-analysis. In addition, their studies did not calculate HWE of the controls for *CYP1A1* MspI genotypes. There may be selection bias or genotyping errors if the control group did not meet HWE. It can lead to misleading results. Moreover, their studies did not assess the credibility of the positive results. The present study has quite a few advantages over the two previous meta-analyses [18,19]: (1) the sample size was much larger, which consists of 23 studies including 5734 cases and 7066 controls; (2) a meta-regression analysis was performed to explore the heterogeneity source; (3) eight genetic models were used; (4) the Venice criteria, FPRP, and BFDP tests were applied to assess the credibility of the positive results. Therefore, our findings should be more credible and convincing.

However, there are still some limitations in the present study. First, language bias could not be avoided because the included studies were written in both English and Chinese. Second, we were not able to perform several important subgroup analyses, such as cancer type, gender, smoking status, and so on. Third, only published articles were selected. Therefore, publication bias may be found as shown in Figure 4. Four, confounding factors did not be controlled such as age, gender, smoking, drinking, and so on, were closely related to affect the results.

These positive findings should be interpreted with caution and results indicate that significant associations may be less-credible, there are no significantly increased LC risk between the combined effects of *GSTM1* present/null and *CYP1A1* MspI polymorphisms.

## Data Availability

All relevant data are presented within the paper.

## Abbreviations

BFDP: Bayesian false discovery probability
CI: confidence interval
CNKI: China National Knowledge Infrastructure
CYP1A1: cytochrome P4501A1
FPRP: false-positive report probability
GSTM1: glutathione-S-transferase M1
HWE: Hardy–Weinberg equilibrium
LC: lung cancer
OR: odds ratio
TSA: trial sequential analysis
